# A systematic review of literature examining the application of a social model of health and wellbeing

**DOI:** 10.1093/eurpub/ckae008

**Published:** 2024-01-26

**Authors:** Rachel Rahman, Caitlin Reid, Philip Kloer, Anna Henchie, Andrew Thomas, Reyer Zwiggelaar

**Affiliations:** Department of Psychology, Aberystwyth University, Aberystwyth, UK; Department of Psychology, Aberystwyth University, Aberystwyth, UK; Hywel Dda University Health Board, St Davids Park, Jobswell Road, Carmarthen, UK; Hywel Dda University Health Board, St Davids Park, Jobswell Road, Carmarthen, UK; Department of Business and Management, Aberystwyth Business School, Aberystwyth University, Aberystwyth, UK; Department of Computer Science, Aberystwyth University, Aberystwyth, UK

## Abstract

**Background:**

Following years of sustained pressure on the UK health service, there is recognition amongst health professionals and stakeholders that current models of healthcare are likely to be inadequate going forward. Therefore, a fundamental review of existing social models of healthcare is needed to ascertain current thinking in this area, and whether there is a need to change perspective on current thinking.

**Method:**

Through a systematic research review, this paper seeks to address how previous literature has conceptualized a social model of healthcare and, how implementation of the models has been evaluated. Analysis and data were extracted from 222 publications and explored the country of origin, methodological approach, and the health and social care contexts which they were set.

**Results:**

The publications predominantly drawn from the USA, UK, Australia, Canada and Europe identified five themes namely: the lack of a clear and unified definition of a social model of health and wellbeing; the need to understand context; the need for cultural change; improved integration and collaboration towards a holistic and person-centred approach; measuring and evaluating the performance of a social model of health.

**Conclusion:**

The review identified a need for a clear definition of a social model of health and wellbeing. Furthermore, consideration is needed on how a model integrates with current models and whether it will act as a descriptive framework or, will be developed into an operational model. The review highlights the importance of engagement with users and partner organizations in the co-creation of a model of healthcare.

## Introduction

Following years of sustained and increasing pressure brought about through inadequate planning and chronic under-resourcing including the unprecedented challenges of the Covid-19 pandemic, the UK NHS is at crisis point.^1^ The incidents of chronic disease continue to increase alongside an ageing population who have more complex health and wellbeing needs, whilst recruitment and retention of staff continue to be insufficient to meet these increased demands.^1^ Furthermore, the Covid-19 pandemic has only served to exacerbate pressures, resulting in delays in; patient presentation,^2^ poor public mental health^3^ strain and burnout amongst workforce.^4^ However, preceding the pandemic there was already recognition of a need for a change to the current biomedical model of care to better prevent and treat the needs of the population.^5^

While it is recognized that demands on the healthcare system are increasing rapidly, the biomedical model used to deal with these issues (which is the current model of healthcare provision in the UK) has largely remained unchanged over the years. The biomedical model takes the perspective that ill-health stems from biological factors and operates on the theory that good health and wellbeing is merely the absence of illness. Application of the model therefore focuses treatment on the management of symptoms and cure of disease from a biological perspective. This suggests that the biomedical approach is mainly reactive in nature and whilst rapid advancements in technology such as diagnostics and robotics have significantly improved patient outcomes and identification of early onset of disease, it does not fully extend into managing the social determinants that can play an important role in the prevention of disease. Therefore, despite its contribution in advancing many areas of biological and health research, the biomedical model has come under increasing scrutiny.^6^ This is in part due to the growing recognition of the impact of those wider social determinants on health, ill-health and wellbeing including physical, mental and social wellbeing which moves the focus beyond individual physical abilities or dysfunction.^7–9^ In order to address these determinants, action needs to be taken through developing policies in a range of non-medical areas such as social, economic and environment so that they regulate the commercial and corporate determinants. In this sense, we can quickly see that the traditional biological model rapidly becomes inadequate. With the current model, health care and clinical staff can do little to affect these determinants and as such can do little to assist the individual patient or society. The efficiency and effectiveness of clinical work will undoubtedly improve if staff have the ability to observe and understand the wider social determinants and consequences of the individual patients’ condition. Therefore, in order to provide a basis for understanding the determinants of disease and arriving at rational treatments and patterns of health care, a medical model must also take into account the patient, the social context in which they live, and a system devised by society to deal with the disruptive effects of illness, that is, the physician’s role and that of the health care system. Models such as Engel’s biopsychosocial model,^9^^,^^10^ the social model of disability, social–ecological models of health^10^^,^^11^ including the World Health Organisation’s framework for action on social determinants of health^8^^,^^9^ are all proposed as attempting to integrate these wider social determinants.

However, the ability of health systems to effectively transition away from a dominant biomedical model to the adoption of a social model of health and care have yet to be fully developed. Responsibility for taking action on these social determinants will need to come from other sectors and policy areas and so future health policy will need to evolve into a more comprehensive and holistic social model of health and wellbeing. Wales’ flagship Wellbeing of Future Generations Act^12^ for instance outlines ways of working towards sustainable development and includes the need to collaborate with society and communities in developing and achieving wellbeing goals. However, developing and implementing an effective operational model that allows multi-stakeholder integration will prove far more difficult to achieve than creating the polices. Furthermore, if the implementation of a robust model of social health is achievable, it’s efficiency, effectiveness and ability to deliver has yet to be proven. Therefore, any future model will need to extend past its conceptual development and provide an ability to manage the complex interactions that will exist between the stakeholders and polices.

Therefore, the use of the term ‘model’ poses its own challenges and debates. Different disciplines attribute differing parameters to what constitutes a model and this in turn may influence the interpretations or expectations surrounding what a model should comprise of or deliver.^13^ According to numerous authors, a model has no ontological category and as such anything from physical entities, theoretical concepts, descriptive frameworks or equations can feasibly be considered a model.^14^ It appears therefore, that much discussion has focussed on the move towards a ‘descriptive’ Social Model of Health and Wellbeing in an attempt to view health more holistically and identify a wider range of determinants that can impact on the health of the population. However, in defining an operational social model of health that can facilitate organizational change, there may be a need to consider a more systems- or process-based approach.

As a result, this review seeks to systematically explore the academic literature in order to better understand how a social model of health and wellbeing is conceptualized, implemented, operationalized and evaluated in health and social care.

The review seeks to address the research questions:

- How is ‘a social model of health and wellbeing’ conceptualized?
- How have social models of health and wellbeing been implemented and evaluated?

## Methods

A systematic search of the literature was carried out between 6 January 2022 and 20 January 2022. Using the search terms shown in table 1, a systematic search was carried out using online databases PsycINFO, ASSIA, IBSS, Medline, Web of Science, CINHAL and SCOPUS. English language and peer-reviewed journals were selected as limiters.

**Table 1:** Search terms

“social model* of care” OR “social model* of health” OR “social model* of healthcare” OR “social model* of health care” OR “social model* of health and wellbeing” OR “social model* of health and wellbeing” OR “social model* of wellbeing” OR “social model* of wellbeing” OR “biopsychosocial model* of care” OR “biopsychosocial model* of health” OR “biopsychosocial model* of healthcare” OR “biopsychosocial model* of health care” OR “biopsychosocial model* of health and wellbeing” OR “biopsychosocial model* of health and wellbeing” OR “biopsychosocial model* of wellbeing” OR “biopsychosocial model* of wellbeing” OR “community model* of care” OR “community model* of health” OR “community model* of healthcare” OR “community model* of health care” OR “community model* of health and wellbeing” OR “community model* of health and wellbeing” OR “community model* of wellbeing” OR “community model* of wellbeing”

### Selection and extraction criteria

**Figure 1:**
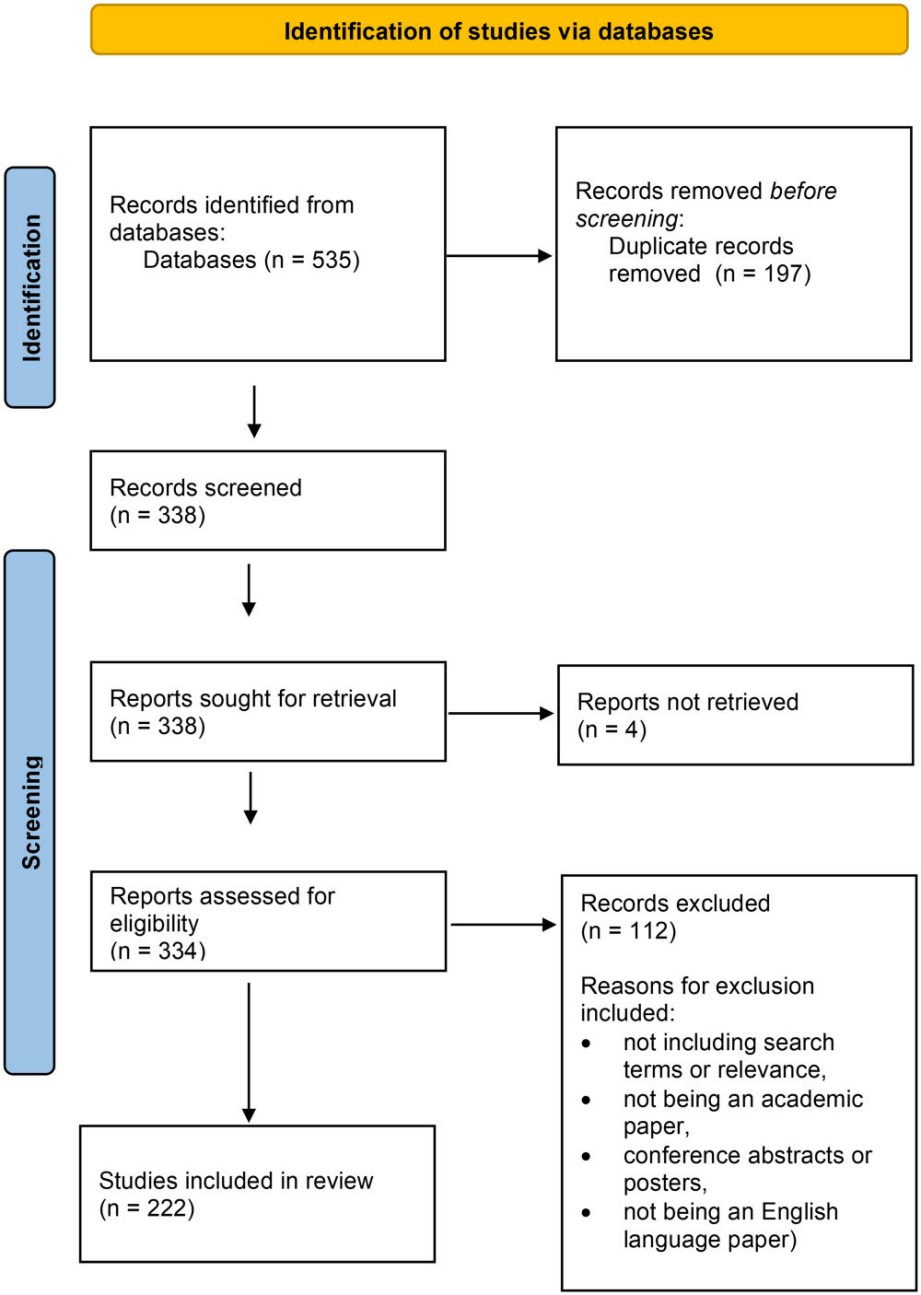
PRISMA flow chart.

The search strategy considered research that explicitly included, framed, or adopted a ‘social model of health and wellbeing’. Each paper was checked for relevance and screened. The authors reviewed the literature using the Preferred Reporting Items for Systematic Reviews and Meta Analysis (PRISMA) method using the updated guidelines from 2020.^15^  Figure 1 represents the process followed.

### Data extraction and analysis

A systematic search of the literature identified 222 eligible papers for inclusion in the final review. A data extraction table was used to extract information regarding location of the research, type of paper (e.g. review, empirical), service of interest and key findings. Quantitative studies were explored with a view to conducting a quantitative meta-analysis; however, given the disparate nature of the outcome measures, and research designs, this was deemed unfeasible. All included papers were coded using NVivo software with the identified research questions in mind, and re-analysed using Thematic Analysis^16^ to explore common themes of relevance.

## Results

The majority of papers were from the USA (34%), with the UK (28%), Australia (16%), Canada (6%) and wider Europe (10%) also contributing to the field. The ‘other’ category (6%) was made up of single papers from other countries. Papers ranged in date from 1983 to 2021 with no noticeable temporal patterns in country of origin, health context or model definition. However, the volume of papers published relating to the social model for healthcare in each decade increased significantly, thus suggesting the increasing research interest towards the social model of healthcare. Table 2 shows the number of publications per decade that were identified from this study.

**Table 2:** Publications identifying social models of healthcare.

Year of publication	Number of publications identifying social models of healthcare
1980s	5
1990s	11
2000	70
2010	87
2020–22	49

**Figure 2:**
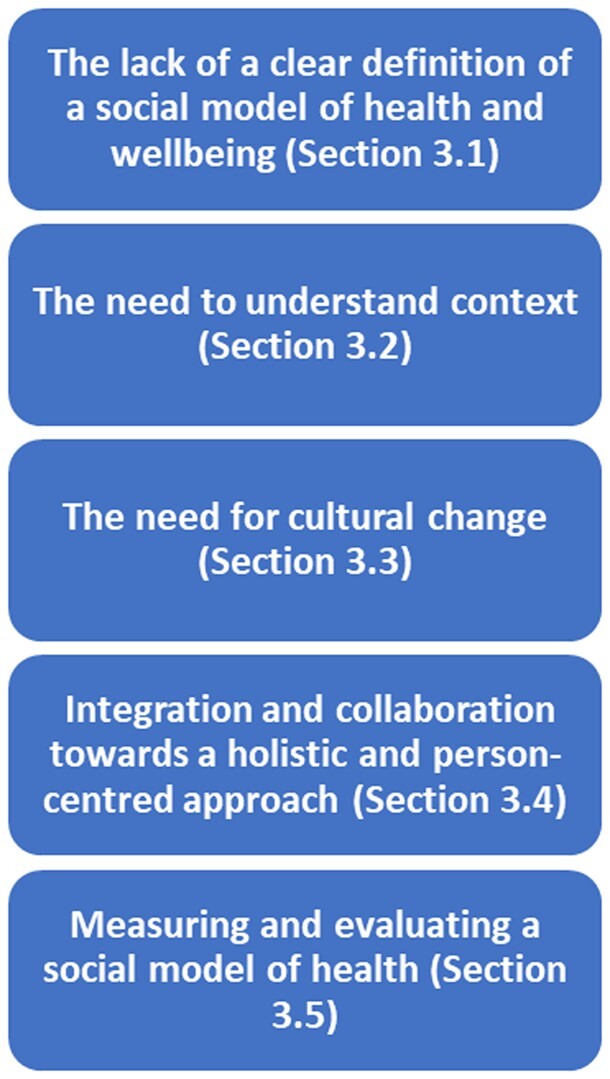
Overview of meta-synthesis themes.

Most of the papers were narrative reviews (*n* = 90) with a smaller number of systematic reviews (*n* = 9) and empirical research studies including qualitative (*n* = 47), quantitative (*n* = 39) and mixed methods (*n* = 14) research. The remaining papers (*n* = 23) comprised small samples of, for example, clinical commentaries, cost effectiveness analysis, discussion papers and impact assessment development papers. The qualitative meta-analysis identified five overarching themes in relation to the research questions, some with underlying sub-themes, which are outlined in figure 2.

### The lack of a clear and unified definition of a social model of health and wellbeing

There was common recognition amongst the papers that a key aim of applying a social model of health and wellbeing was to better address the social determinants of health. Papers identified and reviewed relevant frameworks and models, which they later used to conceptualize or frame their approach when attempting to apply a social model of health. Amongst the most commonly referenced was the WHO’s framework.^17^ Engel’s biopsychosocial model^9^ which was referred to as a seminal framework by many of the researchers. However, once criticism of the biopsychosocial model was its inability to fully address social needs. As a result, a number of papers reported the development of new or enhanced models that used the biopsychosocial model as their underpinning ‘social model’^18^^,^^19^ but then extended their work by including a wider set of social elements in their resulting models.^20^ The Social ecological model,^11^ the Society-Behaviour-Biology Nexus,^21^ and the Environmental Affordances Model are such examples.^22^ Further examples of ‘Social Models’ included the Model of Social Determinants of Health^23^ which framed specific determinants of interest (namely social gradient, stress, early life, social exclusion, work, unemployment, social support, addiction, food and transport). Similarly, Dahlgren and Whitehead’s ‘social model’^10^ illustrates social determinants via a range of influential factors from the individual to the wider cultural and socioeconomic influences. However, none of these papers formally developed a working ‘definition’ of a social model of health and wellbeing, instead applying guiding principles and philosophies associated with a social model to their discussions or interventions.^24^^,^^25^

### The need to understand context

Numerous articles highlight that in order to move towards a social model of health and wellbeing, it is important to understand the context of the environment in which the model will need to operate. This includes balancing the needs of the individual with the resulting model to have been co-created, developed and implemented within the community whilst ensuring that the complexity of interaction between the social determinants of health and their influence on health and wellbeing outcomes are delivered effectively and efficiently.

The literature identified the complex multi-disciplinary nature of a variety of conditions or situations involving medical care. These included issues such as, but not exclusively, chronic pain,^26^ cancer,^27^ older adult care^28^ and dementia,^29^ thus indicating the complex arrangement of medical issues that a model will need to address and, where many authors acknowledged that the frequently used biomedical models failed to fully capture the holistic nature and need of patients. Papers outlined some of the key social determinants of health affecting the specific population of interest in their own context, highlighting the interactions between wider socioeconomic and cultural factors such as poverty, housing, isolation and transport and health and wellbeing outcomes. Interventions that had successfully addressed individual needs and successful embedded services in communities reported improved outcomes for end users and staff in the form of empowerment, agency, education and belonging.^30^ There was also recognition that the transition to more community-based care could be challenging for health and social care providers who were having to work outside of their traditional models of care and accept a certain level of risk.

### The need for cultural change

A number of papers referred to the need for a ‘culture change’ or ‘cultural shift’ in order to move towards a social model of health and wellbeing. Papers identified how ‘culture change models’ were implemented as a way of adapting to a social model. It was recognized that for culture change models to be effective, staff and the general public needed to be fully engaged with the entire move towards a social model, informing and shaping the mechanisms for the cultural shift as well as the application of the model itself.

### Integration and collaboration towards a holistic and person-centred approach

The importance of integration and collaboration between health professionals, (which includes public, private and third sector organizations), services users and patients were emphasized in the ambition to achieve best practice when applying a social model of health and wellbeing. Papers identified the reported benefits of improved collaboration between, and integration of services which included improved continuity of care throughout complex pathways,^31^ improved return to home or other setting on discharge,^25^ and social connectedness.^32^ Numerous papers discussed the importance of multi-disciplinary teams who were able to support individuals beyond the medicalized model.

A number of papers suggested specific professional roles or structures that would be ideal to act as champions or integrators of collaborative services and communities.^25^^,^^33^ These could act as a link between secondary, primary and community level care helping to identify patient needs and supporting the integration of relevant services.

### Measuring and evaluating a social model of health

Individual papers applying and evaluating interventions based on a social model used a variety of methods to evaluate success. Amongst these, some of the most common outcome measures included; general self-report measures of outcomes such as mental health and perceptions of safety,^34^ wellbeing,^35^ life satisfaction and health social networks and support^19^ Some included condition specific self-report outcomes relevant to the condition in question (e.g. pregnancy, anxiety) and pain inventories.^36^ Other papers considered the in-depth experiences of users or service implementers through qualitative techniques such as in-person interviews.^37^^,^^38^

However, the complexity of developing effective methods to evaluate social models of health were recognized. The need to consider the complex interactions between social determinants, and health, wellbeing, economic and societal outcomes posed particular challenges in developing consistency across evaluations that would enable a conclusive evaluation of the benefits of social models to wider health systems and societal health. Some criticized the over-reliance of quantitative and evidence-based practice methods of evaluation highlighting how these could fail to fully capture the complexity of human behaviour and the manner in which their lives could be affected.

## Discussion

The aim of this systematic review was to better understand how a social model of health and wellbeing is conceptualized, implemented and evaluated in health and social care. The review sought to address the research questions identified in the ‘Introduction’ section of this paper.

With regards to the conceptualization of a social model of health and wellbeing, analysis of the literature suggests that whilst the ethos, values and aspirations of achieving a unified model appears to have consensus. However, a fundamental weakness exists in that there is no single unified definition or operational model of a social model of health and wellbeing applied to the health and social care sector. The decision about how best to conceptualize a ‘social model’ is important both in terms of its operational value but also the implication of the associated semantics. However, without a single or unified definition then implementation or further, operationalization of any model will be almost impossible to develop. Furthermore, use of the term ‘social model’ arguably loses site of the biological factors that are clearly relevant in many elements of clinical medicine. Furthermore, there is no clarification in the literature about what would ‘not’ be considered a social model of health and wellbeing, potentially leading to confusion within health and social care sectors when addressing their wider social remit. This raises questions and requires decisions about whether implementation of a social model of health and wellbeing will need to work alongside or replace the existing biomedical approach.

Authors have advocated that a social model provides a way of ‘thinking’ or articulating an organization’s values and culture.^24^ Common elements of the values associated with a social model amongst the papers reviewed included recognition and awareness of the social determinants of health, increased focus on preventative rather than reactive care, and similarly the importance of quality of ‘life’ as opposed to a focus on quality of ‘care’. However, whilst this approach enables individual services to consider how well their own practices align with a social model, the authors suggest that this does not provide large organizations such as the NHS, with multifaceted services and complex internal and external connections and networks, sufficient guidance to enable large scale evaluation or transition to a widespread operational model of a social model of health and wellbeing. This raises questions about what the model should be: whether its function is to support communication of a complex ethos to encourage reflection and engagement of its staff and end users, or to develop the current illustrative framework into a predictive model that can be utilized as an evaluative tool to inform and measure the success of widespread systems change.

Regarding the potential implementation of a future social model of health and wellbeing, none of the papers evaluated the complex widespread organizational implementation of a social model, instead focusing on specific organizational contexts of services such as long-term care in care homes, etc. Despite this, common elements of successful implementation did emerge from the synthesis. This included the need to wholeheartedly engage and be inclusive of end users in policy and practice change to fully understand the complexity of their social worlds and to ensure that changes to practice and policy were ‘developed with’, as opposed to ‘create for’, the wider public. This also involved ensuring that health, social care and wider multi-disciplinary teams were actively included in the process of culture change from an early stage.

### Implications for future research

The analysis identifies that a significant change of mindset and removal of perceived and actual hierarchical structures (that are historically embedded in health and social care structures) amongst both staff and public is needed although, eradicating socially embedded hierarchies will pose significant challenges in practice. Furthermore, the study revealed that many of the models proposed were conceptually underdeveloped and lacked the capability to be operationalized which in turn compromised their ability to be empirically tested. Therefore, in order that a future ‘implementable and operational’ model of social care and wellbeing can be created, further research into organizational behaviours, organizational learning and stakeholder theory (amongst others) applied to the social care and health environment is needed.

#### Towards defining a social model of health and wellbeing

In attempting to conceptualize a definition for a social model of health and wellbeing, it is important to note that the model needs to be sufficiently broad in scope in order to include the prevailing biomedical while also including the need to draw in the social determinants that provide a view and future trajectory towards social health and wellbeing. Therefore, the authors suggest that the ‘preventative’ approach brought by the improvements in the social health determinants (social, cultural, political, environmental**)** need to be balanced effectively with the ‘remedial/preventative’ focus of the biomedical model (and the associated advancements in diagnostics, technology, vaccines, etc), ensuring that a future model drives cultural change; improved integration and collaboration towards a holistic and person-centred approach whilst ensuring engagement with citizens, users, multi-disciplinary teams and partner organizations to ensure that transition towards a social model of health and wellbeing is undertaken.

## Conclusions, recommendations and limitations of the study

Through a comprehensive literature analysis, this paper has provided evidence that advocates a move towards a social model of health and wellbeing. However, the study has predominantly considered mainly literature from the USA, UK, Canada and Australia and therefore is limited in scope at this stage. The authors are aware of the need to consider research undertaken in non-English speaking countries where a considerable body of knowledge also exists and which will add to further discussion about how that work dovetails into this body of literature and, how it aligns with the biomedical perspective. There is a need for complex organizations such as the NHS and allied organizations to agree a working definition of their model of health and wellbeing, whether that be a social model of health and wellbeing, a biopsychosocial model, a combined model, or indeed a new or revised perspective.^39^

One limitation seen of the models within this study is that at a systems level, most models were conceptual models that characterized current systems or conditions and interventions to the current system that result in localized improvements in systems’ performance. However, for meaningful change to occur, a ‘future state’ model may need to focus on a behavioural systems approach allowing modelling of the complete system to take place in order to understand how the elements within the model^40^ behave under different external conditions and how these behaviours affect overall system performance.

Furthermore, considerable work will be required to engage on a more equal footing with the public, health and social care staff as well as wider supporting organizations in developing workable principles and processes that fully embrace the equality of a social model and challenging the ‘power’ imbalances of the current biomedical model.

## Supplementary Material

ckae008_Supplementary_Data

## Data Availability

The datasets generated and/or analysed during the current study are available in the Data Archive at Aberystwyth University and have been included in the supplementary file attached to this submission. A full table of references for studies included in the review will be provided as a supplementary document. The references below refer to citations in the report which are in addition to the included studies of the synthesis. Key points The review identified five themes namely: the lack of a clear definition of a social model of health and wellbeing; the need to understand context; the need for cultural change; improved integration and collaboration towards a holistic and person-centred approach; measuring and evaluating the performance of a social model of health. The review identified a need for organizations to decide on how a social model is to be defined especially at the interfaces between partner organizations and communities. The implications for public policy in this paper highlights the importance of engagement with citizens, users, multi-disciplinary teams and partner organizations to ensure that transition towards a social model of health and wellbeing is undertaken with holistic needs as a central value.
