# Supplementary material for: A systematic review of literature examining the application of a social model of health and wellbeing
Source: Eur J Public Health. 2024 Jan 26;34(3):467–72. doi: 10.1093/eurpub/ckae008 (PMC11161159; doi:10.1093/eurpub/ckae008)
Supplement: ckae008_Supplementary_Data [file ckae008_supplementary_data.docx]

| 1. Abiodun, O. A. (1991). The need for a holistic approach to patient care. East African medical Journal, 68(1), 25-28. |
| --- |
| 1. Abu Ghazaleh, H., Artom, M., & Sturt, J. (2019). A systematic review of community Leg Clubs for patients with chronic leg ulcers. Primary Health Care Research and Development, 20. doi:10.1017/s1463423618000610 |
| 1. Agarwal, G., & Brydges, M. (2018). Effects of a community health promotion program on social factors in a vulnerable older adult population residing in social housing. BMC geriatrics, 18(1), 95. doi:10.1186/s12877-018-0764-9 |
| 1. Balan, S., Majumder, P., Radhakrishnan, R., Wadhwa, R., & Somvanshi, S. (2021). The Networked Computer Metaphor: A Novel Tool for Psychiatric Trainees to Enhance Utility of the Biopsychosocial Model of Health and Illness. Cureus, 13(8), e17395. doi:10.7759/cureus.17395 |
| 1. Baldwin, A., Harvey, C., Willis, E., Ferguson, B., & Capper, T. (2019). Transitioning across professional boundaries in midwifery models of care: A literature review. Women and Birth, 32(3), 195-203. doi:10.1016/j.wombi.2018.08.003 |
| 1. Bambridge, J. (2012). Midwifery care for women with Müllerian anomalies. British Journal of Midwifery, 20(10), 691-696. doi:10.12968/bjom.2012.20.10.691 |
| 1. Barilla, D., Shah, H., & Rawson, R. (2019). Bold leadership is needed for transforming health care. Journal of healthcare leadership, 11, 81-85. doi:10.2147/JHL.S200952 |
| 1. Baum, F., & Freeman, T. (2021). Why Community Health Systems Have Not Flourished in High Income Countries: What the Australian Experience Tells Us. International journal of health policy and management. doi:10.34172/ijhpm.2021.42 |
| 1. Bawden, R., Renyi, R., & Grecs, J. E. T. B. (2018). Research into the effectiveness of the lindsay leg club® model. Wounds UK, 14(4), 36-39. Retrieved from https://www.scopus.com/inward/record.uri?eid=2-s2.0-85054060288&partnerID=40&md5=333ef3cb9e960c5a4f49d9a1d9324d10 |
| 1. Berger, P. (2007). Perspectives on physiotherapy guidelines for chronic low back pain. South African Journal of Physiotherapy, 63(3), 15-21. Retrieved from https://search.ebscohost.com/login.aspx?direct=true&db=cin20&AN=105796036&site=ehost-live |
| 1. Berrios-Rivera, R., Rivero-Vergine, A., & Romero, I. (2008). The Pediatric Cancer Hospitalization Experience: Reality Co-constructed. Journal of Pediatric Oncology Nursing, 25(6), 340-353. doi:10.1177/1043454208323618 |
| 1. Black, M., McKie, L., & Allen, E. (2003). A community development approach to tobacco control. Health Education, 103(2), 68-74. doi:http://dx.doi.org/10.1108/09654280310467681 |
| 1. Blount, A., & Bayona, J. (1994). Toward a system of integrated primary care. Family Systems Medicine, 12(2), 171-182. doi:10.1037/h0089151 |
| 1. Brady, S. S., Bavendam, T. G., Berry, A., Fok, C. S., Gahagan, S., Goode, P. S., . . . Lukacz, E. S. (2018). The Prevention of Lower Urinary Tract Symptoms (PLUS) in girls and women: Developing a conceptual framework for a prevention research agenda. Neurourology and Urodynamics, 37(8), 2951-2964. doi:10.1002/nau.23787 |
| 1. Brand, G., Morrison, P., Down, B., & WestBrook, B. (2014). Scaffolding young Australian women's journey to motherhood: a narrative understanding. Health & Social Care in the Community, 22(5), 497-505. doi:http://dx.doi.org/10.1111/hsc.12106 |
| 1. Brandt, C. (2021). Physiotherapy and pelvic floor health within a contemporary biopsychosocial model of care: From research to education and clinical practice. The South African journal of physiotherapy, 77(1), 1538. doi:10.4102/sajp.v77i1.1538 |
| 1. Brune, K. (2011). Culture change in long term care services: Eden-greenhouse-aging in the community. Educational Gerontology, 37(6), 506-525. doi:10.1080/03601277.2011.570206 |
| 1. Buselli, E. F., & Stuart, E. M. (1999). Influence of psychosocial factors and biopsychosocial interventions on outcomes after myocardial infarction. The Journal of cardiovascular nursing, 13(3), 60-72. doi:10.1097/00005082-199904000-00006 |
| 1. Callahan, L. F. (2016). The History of Patient-Reported Outcomes in Rheumatology. Rheumatic Disease Clinics of North USA, 42(2), 205-+. doi:10.1016/j.rdc.2016.01.012 |
| 1. Camic, P. M., & Chatterjee, H. J. (2013). Museums and art galleries as partners for public health interventions. Perspectives in Public Health, 133(1), 66-71. Retrieved from https://www.proquest.com/scholarly-journals/museums-art-galleries-as-partners-public-health/docview/1314753597/se-2 |
| 1. Cancelliere, C., & Mohammed, R. J. (2019). Brain Drain: Psychosocial Factors Influence Recovery Following Mild Traumatic Brain Injury-3 Recommendations for Clinicians Assessing Psychosocial Factors. Journal of Orthopaedic & Sports Physical Therapy, 49(11), 842-+. doi:10.2519/jospt.2019.8849 |
| 1. Capitman, J. (2003). Effective Coordination of Medical and Supportive Services. Journal of Aging and Health, 15(1), 124-164. doi:10.1177/0898264302239020 |
| 1. Capolongo, S., Lemaire, N., Oppio, A., Buffoli, M., & Le Gall, A. R. (2016). Action planning for healthy cities: the role of multi-criteria analysis, developed in Italy and France, for assessing health performances in land-use plans and urban development projects. Epidemiologia & Prevenzione, 40(3-4), 257-264. doi:10.19191/ep16.3-4.P257.093 |
| 1. Caspar, S., O'Rourke, N., & Gutman, G. M. (2009). The differential influence of culture change models on long-term care staff empowerment and provision of individualized care. Canadian journal on aging = La revue canadienne du vieillissement, 28(2), 165-175. doi:10.1017/S0714980809090138 |
| 1. Cassileth, B. R. (1984). Hospice and the biopsychosocial model of health care: Will hospice be the mechanism for change in the USAn health care system. USAn Journal of Hospice and Palliative Medicine, 1(1), 18-20. doi:10.1177/104990918400100103 |
| 1. Cataldo, I., Azhari, A., Coppola, A., Bornstein, M. H., & Esposito, G. (2019). The Influences of Drug Abuse on Mother-Infant Interaction Through the Lens of the Biopsychosocial Model of Health and Illness: A Review. Frontiers in Public Health, 7. doi:10.3389/fpubh.2019.00045 |
| 1. Chan, W. M., Woo, J., Hui, E., Lau, W. W. Y., Lai, J. C. K., & Lee, D. (2005). A community model for care of elderly people with diabetes via telemedicine. Applied nursing research : ANR, 18(2), 77-81. doi:10.1016/j.apnr.2004.11.002 |
| 1. Chigangaidze, R. K. (2021). Risk Factors and Effects of the Morbus: COVID-19 through the Biopsychosocial Model and Ecological Systems Approach to Social Work Practice. Social Work in Public Health, 36(2), 98-117. doi:10.1080/19371918.2020.1859035 |
| 1. Chin, N., Monroe, A., & Fiscella, K. (2000). Social Determinants of (Un)Healthy Behaviors. Education for Health, 13(3), 317-328. Retrieved from https://www.proquest.com/scholarly-journals/social-determinants-un-healthy-behaviors/docview/2258171998/se-2 |
| 1. Chow, Y. W., & Tsang, H. W. (2007). Biopsychosocial effects of qigong as a mindful exercise for people with anxiety disorders: a speculative review. Journal of Alternative & Complementary Medicine, 13(8), 831-839. doi:10.1089/acm.2007.7166 |
| 1. Christensen, H., Griffiths, K. M., Gulliver, A., Clack, D., Kljakovic, M., & Wells, L. (2008). Models in the delivery of depression care: A systematic review of randomised and controlled intervention trials. Bmc Family Practice, 9. doi:10.1186/1471-2296-9-25 |
| 1. Clarke, J. (2004). Public Health Nursing in Ireland: A Critical Overview. Public Health Nursing, 21(2), 191-198. doi:10.1111/j.0737-1209.2004.021214.x |
| 1. Cohen, A. B., & Koenig, H. G. (2003). RELIGION, RELIGIOSITY AND SPIRITUALITY IN THE BIOPSYCHOSOCIAL MODEL OF HEALTH AND AGEING. Ageing International, 28(3), 215-241. Retrieved from https://search.ebscohost.com/login.aspx?direct=true&db=cin20&AN=104339198&site=ehost-live |
| 1. Compas, B. E., & Boyer, M. C. (2001). Coping and attention: Implications for child health and pediatric conditions. Journal of Developmental and Behavioral Pediatrics, 22(5), 323-333. doi:10.1097/00004703-200110000-00007 |
| 1. Cooke, A., & Coggins, T. (2005). Neighbourhood well-being in Lewisham and Lambeth: the development of a mental well-being impact assessment and indicator toolkit. Journal of Public Mental Health, 4(2), 23-30. doi:http://dx.doi.org/10.1108/17465729200500015 |
| 1. Cooney, T. M., & McClintock, J. (2006). Family members' perspectives on environmental changes in a long-term care facility: Lessons from a case study. Ageing International, 31(1), 71-92. doi:http://dx.doi.org/10.1007/s12126-006-1005-7 |
| 1. Craddock, C., & Reid, M. (1993). STRUCTURE AND STRUGGLE - IMPLEMENTING A SOCIAL MODEL OF A WELL WOMAN CLINIC IN GLASGOW. Social Science & Medicine, 36(1), 67-76. doi:10.1016/0277-9536(93)90306-o |
| 1. Craig, K. D., Holmes, C., Hudspith, M., Moor, G., Moosa-Mitha, M., Varcoe, C., & Wallace, B. (2020). Pain in persons who are marginalized by social conditions. Pain, 161(2), 261-265. doi:10.1097/j.pain.0000000000001719 |
| 1. Crossley, M. L., Nicolson, P., & Owens, G. (2001). Do we need to rethink health psychology? Psychology, Health and Medicine, 6(3), 243-265. Retrieved from https://www.proquest.com/scholarly-journals/do-we-need-rethink-health-psychology/docview/57445491/se-2?accountid=14680 |
| 1. Crowley-Matoka, M., Saha, S., Dobscha, S. K., & Burgess, D. J. (2009). Problems of quality and equity in pain management: Exploring the role of biomedical culture. Pain Medicine, 10(7), 1312-1324. doi:10.1111/j.1526-4637.2009.00716.x |
| 1. Cullen, S. W., & Solomon, P. L. (2013). Family Community Integration and Maternal Mental Health. Administration and Policy in Mental Health and Mental Health Services Research, 40(2), 133-144. doi:http://dx.doi.org/10.1007/s10488-011-0386-4 |
| 1. Dahlen, H. G., Homer, C. S. E., Leap, N., & Tracy, S. K. (2011). From social to surgical: Historical perspectives on perineal care during labour and birth. Women and Birth, 24(3), 105-111. doi:http://dx.doi.org/10.1016/j.wombi.2010.09.002 |
| 1. Damsgaard, J. B., & Angel, S. (2021). Living a meaningful life while struggling with mental health: Challenging aspects regarding personal recovery encountered in the mental health system. International Journal of Environmental Research and Public Health, 18(5), 1-10. doi:10.3390/ijerph18052708 |
| 1. Davies, C. R., Knuiman, M., Wright, P., & Rosenberg, M. (2014). The art of being healthy: A qualitative study to develop a thematic framework for understanding the relationship between health and the arts. Bmj Open, 4(4). doi:10.1136/bmjopen-2014-004790 |
| 1. Davies, C., Knuiman, M., & Rosenberg, M. (2016). The art of being mentally healthy: a study to quantify the relationship between recreational arts engagement and mental well-being in the general population. BMC public health, 16, 15. doi:10.1186/s12889-015-2672-7 |
| 1. Davis, S. L., & Chapa, D. W. (2015). Social Determinants of Health: Knowledge to Effective Action for Change. The Journal for Nurse Practitioners, 11(4), 424-429. doi:http://dx.doi.org/10.1016/j.nurpra.2015.01.029 |
| 1. Davison, T. E., McCabe, M. P., Knight, T., & Mellor, D. (2012). Biopsychosocial factors related to depression in aged care residents. Journal of Affective Disorders, 142(1-3), 290-296. doi:10.1016/j.jad.2012.05.019 |
| 1. de Camargo, O. K. (2011). Systems of care: Transition from the bio‐psycho‐social perspective of the International Classification of Functioning, Disability and Health. Child: Care, Health and Development, 37(6), 792-799. doi:10.1111/j.1365-2214.2011.01323.x |
| 1. de Haan, S. (2021). Bio-psycho-social interaction: an enactive perspective. International Review of Psychiatry, 33(5), 471-477. doi:10.1080/09540261.2020.1830753 |
| 1. de Oliveira, W. (2009). All-around care for patients with Chagas disease: a challenge for the XXI century. Memorias Do Instituto Oswaldo Cruz, 104, 181-186. doi:10.1590/s0074-02762009000900024 |
| 1. Del Piccolo, L., Finset, A., Mellblom, A. V., Figueiredo-Braga, M., Korsvold, L., Zhou, Y., . . . Humphris, G. (2017). Verona Coding Definitions of Emotional Sequences (VR-CoDES): Conceptual framework and future directions. Patient Education and Counseling, 100(12), 2303-2311. doi:10.1016/j.pec.2017.06.026 |
| 1. Dent, E., Davinson, N., & Wilkie, S. (2021). The impact of gastrointestinal conditions on psychosocial factors associated with the biopsychosocial model of health: A scoping review. Applied Psychology: Health and Well-Being. doi:10.1111/aphw.12323 |
| 1. DiLiberto, F. E., Aslan, D. H., Houck, J. R., Ho, B. S., Vora, A. M., & Haddad, S. L. (2020). Overall Health and the Influence of Physical Therapy on Physical Function Following Total Ankle Arthroplasty. Foot & Ankle International, 41(11), 1383-1390. doi:10.1177/1071100720942473 |
| 1. Dobkin, P. L., & Da Costa, D. (2000). Group psychotherapy for medical patients. Psychology, Health & Medicine, 5(1), 87-96. doi:10.1080/135485000106034 |
| 1. Dowrick, C., May, C., Richardson, M., & Bundred, P. (1996). The biopsychosocial model of general practice: rhetoric or reality? The British journal of general practice : the journal of the Royal College of General Practitioners, 46(403), 105-107. Retrieved from https://search.ebscohost.com/login.aspx?direct=true&AuthType=cookie,ip,shib,uid&db=cmedm&AN=8855018&site=ehost-live&scope=site&authtype=shib&custid=s8000044 |
| 1. Driver, C., Oprescu, F., & Lovell, G. P. (2020). An exploration of physiotherapists' perceived benefits and barriers towards using psychosocial strategies in their practice. Musculoskeletal care, 18(2), 111-121. doi:10.1002/msc.1437 |
| 1. Duberstein, Z. T., Brunner, J., Panisch, L. S., Bandyopadhyay, S., Irvine, C., Macri, J. A., . . . O'Connor, T. G. (2021). The Biopsychosocial Model and Perinatal Health Care: Determinants of Perinatal Care in a Community Sample. Frontiers in psychiatry, 12, 746803. doi:10.3389/fpsyt.2021.746803 |
| 1. Eades, G., & Ager, J. (2008). Time Being: difficulties in integrating arts in health. The Journal of the Royal Society for the Promotion of Health, 128(2), 62-67. doi:http://dx.doi.org/10.1177/1466424007087809 |
| 1. Elman, R. J. (2007). The importance of aphasia group treatment for rebuilding community and health. Topics in Language Disorders, 27(4), 300-308. doi:10.1097/01.TLD.0000299884.31864.99 |
| 1. Elman, R. J. (2016). Aphasia centers and the life participation approach to aphasia: A paradigm shift. Topics in Language Disorders, 36(2), 154-167. doi:10.1097/TLD.0000000000000087 |
| 1. Evans, S., Tsao, J. C. I., Sternlieb, B., & Zeltzer, L. K. (2009). Using the Biopsychosocial Model to Understand the Health Benefits of Yoga. Journal of Complementary & Integrative Medicine, 6(1), 64-65. doi:http://dx.doi.org/10.2202/1553-3840.1183 |
| 1. Fairhurst, E. (2005). Theorizing growing and being older: Connecting physical health, well-being and public health. Critical Public Health, 15(1), 27-38. doi:10.1080/09581590500048341 |
| 1. Fisher, M. (2019). A theory of public wellbeing. BMC public health, 19(1). doi:10.1186/s12889-019-7626-z |
| 1. Fisher, M. (2021). Moving Social Policy from Mental Illness to Public Wellbeing. Journal of Social Policy. doi:10.1017/S0047279421000866 |
| 1. Fogel, J. M. D., Vitale, C. M. D., & Peterson, M. M. D. (2008). Finding Common Ground: An Integrative Model of Geriatric Medicine and Geriatric Psychiatry Fellowship Education. Care Management Journals, 9(4), 192-198. doi:http://dx.doi.org/10.1891/1521-0987.9.4.192 |
| 1. Forester, S. (2004). Adopting community development approaches: The Journal of the Health Visitors' Association. Community Practitioner, 77(4), 140-145. Retrieved from https://www.proquest.com/scholarly-journals/adopting-community-development-approaches/docview/213351254/se-2?accountid=14680 |
| 1. Foster, N. E., & Delitto, A. (2011). Embedding Psychosocial Perspectives Within Clinical Management of Low Back Pain: Integration of Psychosocially Informed Management Principles Into Physical Therapist Practice-Challenges and Opportunities. Physical therapy, 91(5), 790-803. doi:10.2522/ptj.20100326 |
| 1. Fried, L. P., Carlson, M. C., Freedman, M., Frick, K. D., Glass, T. A., Hill, J., . . . Zeger, S. (2004). A social model for health promotion for an aging population: initial evidence on the Experience Corps model. Journal of urban health : bulletin of the New York Academy of Medicine, 81(1), 64-78. doi:10.1093/jurban/jth094 |
| 1. Galazka, A. M., Edwards, T., & Harding, K. (2021). Realist evaluation of social outcomes in community care: the application of affordance theory to the Lindsay Leg Clubs. Journal of Critical Realism, 20(3), 280-299. doi:10.1080/14767430.2021.1918969 |
| 1. Gentry Jr, K. K., Snyder, K., & Utley, J. J. (2021). Clinical Utility of the Adapted Biopsychosocial Model: An Initial Validation Through Peer Review. Open Journal of Occupational Therapy (OJOT), 9(2), 1-21. doi:10.15453/2168-6408.1750 |
| 1. Geyh, S., Nick, E., Stirnimann, D., Ehrat, S., Michel, F., Peter, C., & Lude, P. (2012). Self-efficacy and self-esteem as predictors of participation in spinal cord injury-an ICF-based study. Spinal Cord, 50(9), 699-706. doi:10.1038/sc.2012.18 |
| 1. Ghane, A., & Sweeny, K. (2013). Embodied health: a guiding perspective for research in health psychology. Health Psychology Review, 7(sup1), 159. doi:http://dx.doi.org/10.1080/17437199.2012.706988 |
| 1. Giorgi, F., Tramonti, F., & Fanali, A. (2020). A Biosemiotic Approach to the Biopsychosocial Understanding of Disease Adjustment. Biosemiotics, 13(3), 369-383. doi:10.1007/s12304-020-09394-9 |
| 1. Goetz, D. R., & Caron, W. (2005). Systemic Healing: An Ecosystemic Biopsychosocial Integration Applied to Clinical Practice in the Care of Sick Children. Clinical Child Psychology and Psychiatry, 10(1), 53-63. doi:10.1177/1359104505048791 |
| 1. Golden, T. L., & Wendel, M. L. (2020). Public Health's Next Step in Advancing Equity: Re-evaluating Epistemological Assumptions to Move Social Determinants From Theory to Practice. Frontiers in Public Health, 8. doi:10.3389/fpubh.2020.00131 |
| 1. Gomaa, N., Tenenbaum, H., Glogauer, M., & Quiñonez, C. (2019). The Biology of Social Adversity Applied to Oral Health. Journal of dental research, 98(13), 1442-1449. doi:10.1177/0022034519876559 |
| 1. Gordeev, V. S., Maksymowych, W. P., Evers, S., Ament, A., Schachna, L., & Boonen, A. (2010). Role of contextual factors in health-related quality of life in ankylosing spondylitis. Annals of the rheumatic diseases, 69(1), 108-112. doi:10.1136/ard.2008.100164 |
| 1. Gordon, L., Edwards, H., Courtney, M., Finlayson, K., Shuter, P., & Lindsay, E. (2006). A cost-effectiveness analysis of two community models of care for patients with venous leg ulcers. Journal of wound care, 15(8), 348-353. doi:10.12968/jowc.2006.15.8.26942 |
| 1. Green, G., Jackisch, J., & Zamaro, G. (2015). Healthy cities as catalysts for caring and supportive environments. Health Promotion International, 30(Supp 1), 99-107. doi:10.1093/heapro/dav037 |
| 1. Gummidi, B., John, R., Burugina Nagaraja, S., & Tripathy, J. P. (2020). Qualitative enquiry on irregular intake of antihypertensive medications to inform a model of care to improve blood pressure control. Contemporary Nurse: A Journal for the Australian Nursing Profession, 56(5/6), 455-465. doi:10.1080/10376178.2020.1844577 |
| 1. Han, T. Y. P. (2008). A Biopsychosocial Perspective to the Burnout of Korean Workers With Diabetes. USAn Journal of Health Behavior, 32(6), 741-753. doi:http://dx.doi.org/10.5993/AJHB.32.6.18 |
| 1. Harris, E., Barker, C., Burton, K., Lucock, M., & Astin, F. (2020). Self-management support activities in primary care: A qualitative study to compare provision across common health problems. Patient Education and Counseling, 103(12), 2532-2539. doi:10.1016/j.pec.2020.07.003 |
| 1. Haslam, S. A., Haslam, C., Jetten, J., Cruwys, T., & Bentley, S. (2019). Group life shapes the psychology and biology of health: The case for a sociopsychobio model. Social and Personality Psychology Compass, 13(8). doi:10.1111/spc3.12490 |
| 1. Haslam, S. A., Haslam, C., Jetten, J., Cruwys, T., & Bentley, S. V. (2021). Rethinking the nature of the person at the heart of the biopsychosocial model: Exploring social changeways not just personal pathways. Social Science and Medicine, 272. doi:10.1016/j.socscimed.2020.113566 |
| 1. Hauge, H. A., & Hem, H. E. (2011). Developing health promotion education: Mainstreaming or acknowledging tensions in an evolving discipline. Scandinavian Journal of Public Health, 39(supplement 6), 79-84. doi:http://dx.doi.org/10.1177/1403494810384428 |
| 1. Heath, L. (2007). Health impact assessment as a framework for evaluation of local complex projects: The Journal of the Health Visitors' Association. Community Practitioner, 80(7), 30-34. Retrieved from https://www.proquest.com/scholarly-journals/health-impact-assessment-as-framework-evaluation/docview/213307514/se-2?accountid=14680 |
| 1. Hebblethwaite, S. (2013). "I Think That It Could Work But...": Tensions Between the Theory and Practice of Person-Centred and Relationship-Centred Care. Therapeutic Recreation Journal, 47(1), 13-34. Retrieved from https://www.proquest.com/scholarly-journals/i-think-that-could-work-tensions-between-theory/docview/1418164605/se-2?accountid=14680 |
| 1. Hogg, R. R. G. N. B. P., & Hanley, J. P. M. B. R. G. N. (2008). Community development in primary care: opportunities and challenges: The Journal of the Health Visitors' Association. Community Practitioner, 81(1), 22-25. Retrieved from https://www.proquest.com/scholarly-journals/community-development-primary-care-opportunities/docview/213374951/se-2?accountid=14680 |
| 1. James, E. L., Fraser, C., Anderson, K., & Judd, F. (2007). Use of research by the Australian health promotion workforce. Health Education Research, 22(4), 576-587. doi:http://dx.doi.org/10.1093/her/cyl111 |
| 1. Jensen, A. (2018). Mental health recovery and arts engagement. [Mental health recovery]. The Journal of Mental Health Training, Education, and Practice, 13(3), 157-166. doi:http://dx.doi.org/10.1108/JMHTEP-08-2017-0048 |
| 1. Jones, S. C., & Donovan, R. J. (2004). Does theory inform practice in health promotion in Australia? Health Education Research, 19(1), 1-14. Retrieved from https://www.proquest.com/scholarly-journals/does-theory-inform-practice-health-promotion/docview/199522487/se-2 |
| 1. Karunamuni, N., Imayama, I., & Goonetilleke, D. (2021). Pathways to well-being: Untangling the causal relationships among biopsychosocial variables. Social Science and Medicine, 272. doi:10.1016/j.socscimed.2020.112846 |
| 1. Kassie, S. A., Alia, J., & Hyland, L. (2021). Biopsychosocial implications of living with multiple sclerosis: a qualitative study using interpretative phenomenological analysis. Bmj Open, 11(8). doi:10.1136/bmjopen-2021-049041 |
| 1. Keleher, H., & Reiger, K. (2004). Tensions in maternal and child health policy in Victoria: looking back, looking forward. Australian health review : a publication of the Australian Hospital Association, 27(2), 17-26. doi:10.1071/AH042720017 |
| 1. Keller, H. H., Chaudhury, H., Pfisterer, K. J., & Slaughter, S. E. (2018). Development and inter-rater reliability of the Mealtime Scan for long-term care. The Gerontologist, 58(3), e160-e167. doi:10.1093/geront/gnw264 |
| 1. Khoury, P. (2015). Beyond the Biomedical Paradigm: The Formation and Development of Indigenous Community-Controlled Health Organizations in Australia. International journal of health services : planning, administration, evaluation, 45(3), 471-494. doi:10.1177/0020731415584557 |
| 1. Kingsley, A., Hampton, S., Lindsay, E., & Renyi, R. (2017). The Barnstaple Leg Club, Devon. British journal of nursing (Mark Allen Publishing), 26(7), 426-427. doi:10.12968/bjon.2017.26.7.426 |
| 1. Klein, H. (2004). Health inequality, social exclusion and neighbourhood renewal: Can place-based renewal improve the health of disadvantaged communities? Australian Journal of Primary Health, 10(3), 110-119. doi:10.1071/PY04054 |
| 1. Knapp, M., Funk, M., Curran, C., Prince, M., Grigg, M., & McDaid, D. (2006). Economic barriers to better mental health practice and policy. Health Policy and Planning, 21(3), 157-170. Retrieved from https://www.proquest.com/scholarly-journals/economic-barriers-better-mental-health-practice/docview/210781159/se-2 |
| 1. Knibb, R. C., Cortes, A., Barnes, C., & Stalker, C. (2016). Validation of the English Version of the Scale for Psychosocial Factors in Food Allergy and the Relationship with Mental Health, Quality of Life, and Self-Efficacy. Journal of Allergy, 2016. doi:10.1155/2016/4850940 |
| 1. Kümpers, S., Mur, I., Maarse, H., & van Raak, A. (2005). A Comparative Study of Dementia Care in England and the Netherlands Using Neo-Institutionalist Perspectives. Qualitative Health Research, 15(9), 1199-1230. doi:10.1177/1049732305276730 |
| 1. Laughlin, S., Nandwani, R., Ilett, R., & Bigrigg, A. (2001). The Sandyford Initiative: creating added value to health and health care. Health bulletin, 59(4), 238-243. Retrieved from https://www.scopus.com/inward/record.uri?eid=2-s2.0-0037512403&partnerID=40&md5=fb0be7b2a645a7e72847b28da564b7cc |
| 1. Law, D. D., Crane, D. R., & Berge, J. M. (2003). The influence of individual, marital, and family therapy on high utilizers of health care. Journal of Marital and Family Therapy, 29(3), 353-363. doi:http://dx.doi.org/10.1111/j.1752-0606.2003.tb01212.x |
| 1. Lehman, B. J., David, D. M., & Gruber, J. A. (2017). Rethinking the biopsychosocial model of health: Understanding health as a dynamic system. Social and Personality Psychology Compass, 11(8), 1-17. doi:10.1111/spc3.12328 |
| 1. Lindsay, E. (2008). The Leg Club® Model: Promoting the health of patients' lower limbs through collaborative working. Wounds UK, 4(2), 49-60. Retrieved from https://www.scopus.com/inward/record.uri?eid=2-s2.0-48249110265&partnerID=40&md5=a2ca95ccf29338791e59af0c00342d01 |
| 1. Lindsay, E. (2010). Leg Clubs®: A clinically and cost-effective approach to lower limb management. British Journal of Community Nursing, 15(6 SUPPL.), S16-S23. doi:10.12968/bjcn.2010.15.sup4.48379 |
| 1. Listopad, I. W., Michaelsen, M. M., Werdecker, L., & Esch, T. (2021). Bio-Psycho-Socio-Spirito-Cultural Factors of Burnout: A Systematic Narrative Review of the Literature. Frontiers in Psychology, 12. doi:10.3389/fpsyg.2021.722862 |
| 1. Lonargain, D. O., Brannigan, D., & Murray, C. (2017). The experience of receiving a kidney transplant from a deceased donor: Implications for renal services. Psychology & Health, 32(2), 204-220. doi:10.1080/08870446.2016.1254214 |
| 1. Longo, D. J., & Clum, G. A. (1989). Psychosocial factors affecting genital herpes recurrences: Linear vs mediating models. Journal of Psychosomatic Research, 33(2), 161-166. doi:10.1016/0022-3999(89)90043-3 |
| 1. Lymbery, M. (2006). United We Stand? Partnership Working in Health and Social Care and the Role of Social Work in Services for Older People. British Journal of Social Work, 36(7), 1119-1134. doi:http://dx.doi.org/10.1093/bjsw/bch348 |
| 1. Madani, S., Parikh, S., Madani, R. S., & Krasaelap, A. (2017). Long-Term Study of Children With ROME III Functional Gastrointestinal Disorders Managed Symptomatically in a Biopsychosocial Model. Gastroenterology research, 10(2), 84-91. doi:10.14740/gr798w |
| 1. Maddux, M. H., Bass, J. A., Geraghty-Sirridge, C., Carpenter, E., & Christenson, K. (2013). Assessing psychosocial functioning among youth with newly diagnosed inflammatory bowel disease (IBD): An interdisciplinary clinic approach. Clinical Practice in Pediatric Psychology, 1(4), 333-343. doi:10.1037/cpp0000037 |
| 1. Mahnken, J. E. (2001). Rural nursing and health care reforms: building a social model of health. Rural and remote health, 1(1), 104. Retrieved from https://search.ebscohost.com/login.aspx?direct=true&AuthType=cookie,ip,shib,uid&db=cmedm&AN=15869371&site=ehost-live&scope=site&authtype=shib&custid=s8000044 |
| 1. Markovic, M., Manderson, L., & Kelaher, M. (2002). The health of immigrant women: Queensland women from the former Yugoslavia. Journal of Immigrant Health, 4(1), 5-15. doi:10.1023/A:1013003126561 |
| 1. Martinez, I. L., Frick, K., Glass, T. A., Carlson, M., Tanner, E., Ricks, M., & Fried, L. P. (2006). Engaging older adults in high impact volunteering that enhances health: recruitment and retention in The Experience Corps Baltimore. Journal of urban health : bulletin of the New York Academy of Medicine, 83(5), 941-953. doi:10.1007/s11524-006-9058-1 |
| 1. Masters, K. S. (2006). Recurrent Abdominal Pain, Medical Intervention, and Biofeedback: What Happened to the Biopsychosocial Model? Applied Psychophysiology and Biofeedback, 31(2), 155-165. doi:10.1007/s10484-006-9016-4 |
| 1. Matthews, J., Zoffness, R., & Becker, D. (2021). Integrative pediatric pain management: Impact & implications of a novel interdisciplinary curriculum. Complementary Therapies in Medicine, 59, N.PAG-N.PAG. doi:10.1016/j.ctim.2021.102721 |
| 1. McAllister, C. L., & Silverman, M. A. (1999). Community formation and community roles among persons with Alzheimer's disease: a comparative study of experiences in a residential Alzheimer's facility and a traditional nursing home. Qualitative Health Research, 9(1), 65-85. doi:10.1177/104973299129121703 |
| 1. McBride, J., Block, A., & Russo, A. (2017). An integrated healthcare service for asylum seekers and refugees in the South-Eastern Region of Melbourne: Monash Health Refugee Health and Wellbeing. Australian Journal of Primary Health, 23(4), 323-328. doi:http://dx.doi.org/10.1071/PY16092 |
| 1. McCullough, K., Bayes, S., Whitehead, L., Williams, A., & Cope, V. (2021). We say we are doing primary health care but we're not: Remote area nurses' perspectives on the challenges of providing primary health care services. Collegian, 28(5), 534-540. doi:10.1016/j.colegn.2021.02.006 |
| 1. McKenzie, M. (2013). The lindsay leg club: Supporting the NHS to provide leg ulcer care. British Journal of Community Nursing, 18(6 SUPPL), S16-S20. doi:10.12968/bjcn.2013.18.sup6.s16 |
| 1. McLeod, A., Baker, D., & Black, M. (2006). Investigating the nature of formal social support provision for young mothers in a city in the North West of England. Health & Social Care in the Community, 14(6), 453-464. doi:http://dx.doi.org/10.1111/j.1365-2524.2006.00625.x |
| 1. McMurray, R. (2006). From partition to partnership: Managing collaboration within a curative framework for NHS care. The International Journal of Public Sector Management, 19(3), 238-249. doi:http://dx.doi.org/10.1108/09513550610658204 |
| 1. McNeill, J. A., & Reiger, K. M. (2015). Rethinking prenatal care within a social model of health: An exploratory study in Northern Ireland. Health Care for Women International, 36(1), 5-25. doi:10.1080/07399332.2014.900061 |
| 1. Medich, C., Stuart, E. M., Deckro, J. P., & Friedman, R. (1991). Psychophysiologic control mechanisms in ischemic heart disease: the mind-heart connection. Journal of Cardiovascular Nursing, 5(4), 10-26. Retrieved from https://search.ebscohost.com/login.aspx?direct=true&db=cin20&AN=107504304&site=ehost-live |
| 1. Mehra, R., Boyd, L. M., Magriples, U., Kershaw, T. S., Ickovics, J. R., & Keene, D. E. (2020). Black pregnant women 'get the most judgment': A qualitative study of the experiences of Black women at the intersection of race, gender, and pregnancy. Women's Health Issues, 30(6), 484-492. doi:10.1016/j.whi.2020.08.001 |
| 1. Mescouto, K., Olson, R. E., Hodges, P. W., & Setchell, J. (2020). A critical review of the biopsychosocial model of low back pain care: time for a new approach? Disability and Rehabilitation. doi:10.1080/09638288.2020.1851783 |
| 1. Meyer, J. A. (2007). Strategies for the long-term treatment of schizophrenia: Real-world lessons from the CATIE trial. Journal of Clinical Psychiatry, 68, 28-33. Retrieved from <Go to ISI>://WOS:000244074600005 |
| 1. More, K. R., Quigley-McBride, A., Clerke, A. S., & More, C. (2019). Do measures of country-level safety predict individual-level health outcomes? Social Science & Medicine, 225, 128-138. doi:10.1016/j.socscimed.2019.02.022 |
| 1. Moser, N. L., Plante, W. A., LeLeiko, N. S., & Lobato, D. J. (2014). Integrating behavioral health services into pediatric gastroenterology: A model of an integrated health care program. Clinical Practice in Pediatric Psychology, 2(1), 1-12. doi:10.1037/cpp0000046 |
| 1. Mousavi, S. J., van Dieen, J. H., & Anderson, D. E. (2019). Low back pain: Moving toward mechanism-based management. Clinical Biomechanics, 61, 190-191. doi:10.1016/j.clinbiomech.2018.12.010 |
| 1. Mullins, L., Skemp, L. E., & Maas, M. L. (2016). Community Models of Care: A Scoping Review. Journal of gerontological nursing, 42(12), 12-20. doi:10.3928/00989134-20161110-05 |
| 1. Nair, D., Cukor, D., Taylor, W. D., & Cavanaugh, K. L. (2021). Applying A Biopsychosocial Framework to Achieve Durable Behavior Change in Kidney Disease. Seminars in nephrology, 41(6), 487-504. doi:10.1016/j.semnephrol.2021.10.002 |
| 1. Negri, A., Zamin, C., Parisi, G., Paladino, A., & Andreoli, G. (2021). Analysis of general practitioners’ attitudes and beliefs about psychological intervention and the medicine-psychology relationship in primary care: Toward a new comprehensive approach to primary health care. Healthcare (Switzerland), 9(5). doi:10.3390/healthcare9050613 |
| 1. Németh, R., Sik, D., & Katona, E. (2021). The asymmetries of the biopsychosocial model of depression in lay discourses - Topic modelling online depression forums. SSM - Population Health, 14. doi:10.1016/j.ssmph.2021.100785 |
| 1. Nevin, P. E., Frey, S., Lipira, L., Endeshaw, M., Niemann, L., Kerani, R. P., & Rao, D. (2018). 'You are always hiding It's the worst way to live' exploring stigma in African immigrants living with HIV in a large northwest US metropolitan area. JANAC: Journal of the Association of Nurses in AIDS Care, 29(3), 417-425. Retrieved from https://search.ebscohost.com/login.aspx?direct=true&AuthType=cookie,ip,shib,uid&db=psyh&AN=2018-19045-015&site=ehost-live&scope=site&authtype=shib&custid=s8000044 |
| 1. Newburn, M. (2012). The best of both worlds -- Parents' motivations for using an alongside birth centre from an ethnographic study. Midwifery, 28(1), 61-66. doi:http://dx.doi.org/10.1016/j.midw.2010.10.014 |
| 1. Ngune, I., Kalembo, F., Loessl, B., & Kivuti-Bitok, L. W. (2020). Biopsychosocial risk factors and knowledge of cervical cancer among young women: A case study from Kenya to inform HPV prevention in Sub-Saharan Africa. PLoS ONE, 15(8 August). doi:10.1371/journal.pone.0237745 |
| 1. Nilsson, E., Festin, K., Lowén, M., & Kristenson, M. (2019). Sf-36 predicts 13-year chd incidence in a middle-aged swedish general population. Quality of Life Research: An International Journal of Quality of Life Aspects of Treatment, Care & Rehabilitation. doi:10.1007/s11136-019-02362-y |
| 1. Ning, L. (2010). Building a 'user driven' mental health system. Advances in Mental Health, 9(2), 112-115. doi:http://dx.doi.org/10.5172/jamh.9.2.112 |
| 1. Novack, D. H., Cameron, O., Epel, E., Ader, R., Waldstein, S. R., Levenstein, S., . . . Wainer, A. R. (2007). Psychosomatic medicine: the scientific foundation of the biopsychosocial model. Academic psychiatry : the journal of the USAn Association of Directors of Psychiatric Residency Training and the Association for Academic Psychiatry, 31(5), 388-401. doi:10.1176/appi.ap.31.5.388 |
| 1. Oberski, I. M., Carter, D. E., Gray, M., & Ross, J. (1999). The community gerontological nurse: themes from a needs analysis. Journal of Advanced Nursing, 29(2), 454-462. doi:10.1046/j.1365-2648.1999.00907.x |
| 1. Patel, N. K., Akkihebbalu, S., Espinoza, S. E., & Chiodo, L. K. (2011). Perceptions of a community-based yoga intervention for older adults. Activities, Adaptation and Aging, 35(2), 151-163. doi:10.1080/01924788.2011.574256 |
| 1. Pavlovic, N. V., Gilotra, N. A., Lee, C. S., Ndumele, C., Mammos, D., Dennisonhimmelfarb, C., & AbshireSaylor, M. (2021). Fatigue in Persons With Heart Failure: A Systematic Literature Review and Meta-Synthesis Using the Biopsychosocial Model of Health. Journal of Cardiac Failure. doi:10.1016/j.cardfail.2021.07.005 |
| 1. Penelope Fay, M., & Pattison, P. E. (2012). Organizational culture, intersectoral collaboration and mental health care. Journal of Health Organization and Management, 26(1), 32-59. doi:http://dx.doi.org/10.1108/14777261211211089 |
| 1. Pinnock, D. (2002). Pain management. Effective services for the care of patients with back pain. Professional Nurse, 17(7), 422-424. Retrieved from https://search.ebscohost.com/login.aspx?direct=true&db=cin20&AN=106914627&site=ehost-live |
| 1. Piterman, L., Zimmet, H., Krum, H., Tonkin, A., & Yallop, J. (2005). Chronic heart failure: optimising care in general practice. Australian Family Physician, 34(7), 547-553. Retrieved from https://search.ebscohost.com/login.aspx?direct=true&db=cin20&AN=106429881&site=ehost-live |
| 1. Podgorski, C. A., Anderson, S. D., & Parmar, J. (2021). A Biopsychosocial-Ecological Framework for Family-Framed Dementia Care. Frontiers in psychiatry, 12. doi:10.3389/fpsyt.2021.744806 |
| 1. Priest, J. B. (2019). Examining differentiation of self as a mediator in the biobehavioral family model. Journal of Marital and Family Therapy, 45(1), 161-175. doi:10.1111/jmft.12301 |
| 1. Priest, J. B., Smith, S. M., Woods, S. B., & Roberson, P. N. E. (2020). Discrimination, family emotional climate, and African USAn health: An application of the BBFM. Journal of Family Psychology, 34(5), 598-609. doi:10.1037/fam0000621 |
| 1. Quilgars, D. (2003). Community development with a care and support agenda: Is it achievable? Housing, Care and Support, 6(4), 4-9. doi:http://dx.doi.org/10.1108/14608790200300022 |
| 1. Ra, J. S., & Jeong, Y. H. (2020). Psychosocial factors associated with smoking cessation attempts in Korean high school students who engage in intermittent and light smoking. Child Health Nursing Research, 26(1), 1-10. doi:10.4094/chnr.2020.26.1.1 |
| 1. Ra, J. S., & Kim, H. (2021). Combined effects of unhealthy lifestyle behaviors on metabolic syndrome among postmenopausal women. Healthcare (Switzerland), 9(7). doi:10.3390/healthcare9070848 |
| 1. Randall, S., & Neubeck, L. (2016). What's in a name? Concordance is better than adherence for promoting partnership and self-management of chronic disease. Australian Journal of Primary Health, 22(3), 181-184. doi:http://dx.doi.org/10.1071/PY15140 |
| 1. Rankin, D., Truman, J., Backett-Milburn, K., Platt, S., & Petticrew, M. (2006). The contextual development of healthy living centres services: an examination of food-related initiatives. Health & place, 12(4), 644-655. doi:10.1016/j.healthplace.2005.08.013 |
| 1. Reaney, M., Eichorst, B., & Gorman, P. (2012). From Acorns to Oak Trees: The Development and Theoretical Underpinnings of Diabetes Conversation Map Education Tools. Diabetes Spectrum, 25(2), 111-116. doi:10.2337/diaspect.25.2.111 |
| 1. Reeve, C., Banfield, S., Thomas, A., Reeve, D., & Davis, S. (2016). Community outreach midwifery-led model improves antenatal access in a disadvantaged population. The Australian journal of rural health, 24(3), 200-206. doi:10.1111/ajr.12249 |
| 1. Reinhardt, G. Y., Vidovic, D., & Hammerton, C. (2021). Understanding loneliness: a systematic review of the impact of social prescribing initiatives on loneliness. Perspectives in Public Health, 141(4), 204-213. doi:http://dx.doi.org/10.1177/1757913920967040 |
| 1. Richardson, C. A., & Rabiee, F. (2001). A question of access: an exploration of the factors that influence the health of young males aged 15 to 19 living in Corby and their use of health care services. Health Education Journal, 60(1), 3-16. Retrieved from https://search.ebscohost.com/login.aspx?direct=true&db=cin20&AN=106938354&site=ehost-live |
| 1. Robinson, K., Kennedy, N., & Harmon, D. (2011). Review of occupational therapy for people with chronic pain. Australian Occupational Therapy Journal, 58(2), 74-81. doi:10.1111/j.1440-1630.2010.00889.x |
| 1. Robinson, L. M., Francis, L., Simpson, C., & Rutledge, R. (2006). Raising awareness of psychology in physicians through a Web-based self-change program. Professional Psychology-Research and Practice, 37(5), 477-480. doi:10.1037/0735-7028.37.5.477 |
| 1. Robles, B., Kuo, T., & Tobin, C. S. T. (2021). What are the relationships between psychosocial community characteristics and dietary behaviors in a racially/ethnically diverse urban population in los angeles county? International Journal of Environmental Research and Public Health, 18(18). doi:10.3390/ijerph18189868 |
| 1. Roddy, E., Zwierska, I., Jordan, K. P., Dawes, P., Hider, S. L., Packham, J., . . . Hay, E. M. (2013). Musculoskeletal clinical assessment and treatment services at the primary-secondary care interface: an observational study. British Journal of General Practice, 63(607), E141-E148. doi:10.3399/bjgp13X663109 |
| 1. Rogers, N. (2008). From "Treatment" to "Care": Developing youth drug treatment services using a social model of health. Australian Journal of Primary Health, 14(1), 52-58. doi:10.1071/py08007 |
| 1. Rogers, R. W. (1983). Preventive Health Psychology: An Interface of Social and Clinical Psychology. Journal of Social and Clinical Psychology, 1(2), 120-127. doi:http://dx.doi.org/10.1521/jscp.1983.1.2.120 |
| 1. Rolland, J. S., & Walsh, F. (2005). Systemic Training for Healthcare Professionals: The Chicago Center for Family Health Approach. Family Process, 44(3), 283-301. doi:http://dx.doi.org/10.1111/j.1545-5300.2005.00060.x |
| 1. Rolland, J. S., & Williams, J. K. (2005). Toward a Biopsychosocial Model for 21st-Century Genetics. Family Process, 44(1), 3-24. doi:http://dx.doi.org/10.1111/j.1545-5300.2005.00039.x |
| 1. Roussy, V., Russell, G., Livingstone, C., & Riley, T. (2021). Mergers may enhance the legitimacy of community health organisations in neoliberal environments. Journal of Health Organization and Management, 35(6), 717-732. doi:10.1108/jhom-04-2020-0160 |
| 1. Russell, R. (2013). The rationale for primary spine care employing biopsychosocial, stratified and diagnosis-based care-pathways at a chiropractic college public clinic: a literature review. Chiropractic & manual therapies, 21(1), 19. doi:10.1186/2045-709X-21-19 |
| 1. Russo, D. C., & Tarbell, S. E. (1984). Child health psychology: Emerging responsibilities of the pediatric health psychologist. Clinical Psychology Review, 4(5), 495-502. doi:10.1016/0272-7358(84)90040-0 |
| 1. Salole, E. G. (1994). Are the 'medical' and 'social' models of health fundamentally incompatible in pharmacy practice? A critique. Journal of Social and Administrative Pharmacy, 11(1), 1-6. Retrieved from https://www.scopus.com/inward/record.uri?eid=2-s2.0-0028260752&partnerID=40&md5=d5d2836264e7f55c0b779066bd50ec5b |
| 1. Samoborec, S., Ruseckaite, R., Ayton, D., & Evans, S. (2018). Biopsychosocial factors associated with non-recovery after a minor transport-related injury: A systematic review. PLoS ONE, 13(6). doi:10.1371/journal.pone.0198352 |
| 1. Sanders, T., Foster, N. E., Bishop, A., & Ong, B. N. (2013). Biopsychosocial care and the physiotherapy encounter: physiotherapists' accounts of back pain consultations. Bmc Musculoskeletal Disorders, 14. doi:10.1186/1471-2474-14-65 |
| 1. Santiago Delefosse, M. (2011). An embodied‐socio‐psychological perspective in health psychology? Social and Personality Psychology Compass, 5(5), 220-230. doi:10.1111/j.1751-9004.2011.00345.x |
| 1. Saylor, J., & Friedmann, E. (2015). Biopsychosocial contributors to metabolic syndrome: A secondary analysis of 2007-2010 National Health and Nutrition Examination Survey data. Nursing Research, 64(6), 434-443. doi:10.1097/NNR.0000000000000121 |
| 1. Schemitsch, C., & Nauth, A. (2020). Psychological factors and recovery from trauma. Injury, 51 Suppl 2, S64-S66. doi:10.1016/j.injury.2019.10.081 |
| 1. Schoeb, V. (2016). Healthcare Service in Hong Kong and its Challenges: The Role of Health Professionals within a Social Model of Health. China Perspectives(4), 51-58. Retrieved from https://www.proquest.com/scholarly-journals/healthcare-service-hong-kong-challenges-role/docview/1854202176/se-2?accountid=14680 |
| 1. Seyer, F., Witt, J.-A., Taube, J., & Helmstaedter, C. (2018). The efficacy of a short-term multidisciplinary epilepsy program. Epilepsy & Behavior, 86, 98-101. doi:10.1016/j.yebeh.2018.06.017 |
| 1. Shannon, M. T. (1989). Health promotion and illness prevention: A biopsychosocial perspective. Health & Social Work, 14(1), 32-40. Retrieved from https://search.ebscohost.com/login.aspx?direct=true&AuthType=cookie,ip,shib,uid&db=psyh&AN=1989-23152-001&site=ehost-live&scope=site&authtype=shib&custid=s8000044 |
| 1. Sharp, S., Smedema, S. M., Friefeld, R., & Thompson, K. (2016). Evaluation of a biopsychosocial model of life satisfaction in individuals with spinal cord injuries. Journal of Rehabilitation, 82(4), 38-47. Retrieved from https://www.scopus.com/inward/record.uri?eid=2-s2.0-85014405928&partnerID=40&md5=112cf5542e803862e5e62e2fe99b8c96 |
| 1. Sharrad, K. J., Kopsaftis, Z. A., Carson-Chahhoud, K. V., & Stallman, H. M. (2021). The modifiable biopsychosocial drivers of psychological distress for adolescents with asthma: Implications for Clinical Care. Paediatric Respiratory Reviews. doi:10.1016/j.prrv.2021.07.005 |
| 1. Simovska, V., & Carlsson, M. (2012). Health-promoting changes with children as agents: Findings from a multiple case study research. Health Education, 112(3), 292-304. doi:10.1108/09654281211217803 |
| 1. Smedema, S. M. (2017). Evaluation of a concentric biopsychosocial model of well-being in persons with spinal cord injuries. Rehabilitation psychology, 62(2), 186-197. doi:10.1037/rep0000150 |
| 1. Smith, R. C. (2021). Making the biopsychosocial model more scientific—its general and specific models. Social Science and Medicine, 272. doi:10.1016/j.socscimed.2020.113568 |
| 1. Snape, S. (2003). Health and local government partnerships: The local government policy context. Local Government Studies, 29(3), 73-98. doi:10.1080/03003930308559380 |
| 1. Stephen-Haynes, J. (2010). The Leg Club model: A survey of staff and members' perceptions of this model of care. Journal of wound care, 19(9), 380-387. doi:10.12968/jowc.2010.19.9.78220 |
| 1. Stuart, K., Faghy, M. A., Bidmead, E., Browning, R., Roberts, C., Grimwood, S., & Winn-Reed, T. (2020). A biopsychosocial framework for recovery from COVID-19. International Journal of Sociology and Social Policy, 40(9-10), 1021-1039. doi:10.1108/IJSSP-07-2020-0301 |
| 1. Suls, J., & Rothman, A. (2004). Evolution of the Biopsychosocial Model: Prospects and Challenges for Health Psychology. Health Psychology, 23(2), 119-125. doi:10.1037/0278-6133.23.2.119 |
| 1. Sun, S., & Ching, A. H. (2021). Social systems matter: Precision medicine, public health, and the medical model. East Asian Science Technology and Society-an International Journal, 15(4), 439-466. doi:10.1080/18752160.2021.1938440 |
| 1. Symon, A., Pringle, J., Downe, S., Hundley, V., Lee, E., Lynn, F., . . . Alderdice, F. (2017). Antenatal care trial interventions: A systematic scoping review and taxonomy development of care models. BMC Pregnancy and Childbirth, 17(1). doi:10.1186/s12884-016-1186-3 |
| 1. Taggart, L., McMillan, R., & Lawson, A. (2008). Women with and without intellectual disability and psychiatric disorders: An examination of the literature. Journal of Intellectual Disabilities, 12(3), 191-211. doi:10.1177/1744629508095323 |
| 1. Tan, S. T., Quek, R. Y. C., Haldane, V., Koh, J. J. K., Han, E. K. L., Ong, S. E., . . . Legido-Quigley, H. (2019). The social determinants of chronic disease management: perspectives of elderly patients with hypertension from low socio-economic background in Singapore. International journal for equity in health, 18(1), 1. doi:10.1186/s12939-018-0897-7 |
| 1. Tan, S.-Y., & Haining, R. (2016). Crime victimization and the implications for individual health and wellbeing: A Sheffield case study. Social Science & Medicine, 167, 128-139. doi:10.1016/j.socscimed.2016.08.018 |
| 1. Taylor, A. M., Teijlingen, E. v., Ryan, K. M., & Alexander, J. (2019). 'Scrutinised, judged and sabotaged': A qualitative video diary study of first-time breastfeeding mothers. Midwifery, 75, 16-23. doi:10.1016/j.midw.2019.04.004 |
| 1. Teresa, M. T., Guss, C. D., & Boyd, L. (2021). Thriving during COVID-19: Predictors of psychological well-being and ways of coping. PLoS ONE, 16(3 March). doi:10.1371/journal.pone.0248591 |
| 1. Thomson, H., Petticrew, M., & Morrison, D. (2001). Health effects of housing improvement: Systematic review of intervention studies: BMJ. British Medical Journal, 323(7306), 187-190. Retrieved from https://www.proquest.com/scholarly-journals/health-effects-housing-improvement-systematic/docview/204043293/se-2 |
| 1. Topolski, S. (2009). Understanding health from a complex systems perspective. Journal of Evaluation in Clinical Practice, 15(4), 749-754. doi:10.1111/j.1365-2753.2009.01227.x |
| 1. Townsend, L., Gray, J., & Forber, J. (2016). New ways of seeing: Nursing students' experiences of a pilot service learning program in Australia. Nurse Education in Practice, 16(1), 60-65. doi:http://dx.doi.org/10.1016/j.nepr.2015.08.004 |
| 1. Tripp, S. (1997). What contribution, if any, can social construction theories make to understanding the experience of disabling conditions? Journal of Orthopaedic Nursing, 1(1), 17-20. doi:10.1016/S1361-3111(97)80050-7 |
| 1. Truman, J., Rankin, D., Backett-Milburn, K., & Platt, S. (2007). Drop-in services: Findings from an evaluation of the Healthy Living Centre programme in Scotland. Health Education Journal, 66(1), 22-31. doi:10.1177/0017896907069368 |
| 1. Truong, M., Gibbs, L., Paradies, Y., Priest, N., & Tadic, M. (2017). Cultural competence in the community health context: ‘we don’t have to reinvent the wheel’. Australian Journal of Primary Health, 23(4), 342-347. doi:http://dx.doi.org/10.1071/PY16073 |
| 1. Tucker, C. M., Ferdinand, L. A., Mirsu-Paun, A., Herman, K. C., Delgado-Romero, E., Van Den Berg, J. J., & Jones, J. D. (2007). The Roles of Counseling Psychologists in Reducing Health Disparities. The Counseling Psychologist, 35(5), 650-678. doi:10.1177/0011000007301687 |
| 1. Valsecchi, R., Anderson, N., Balta, M. E., & Harrison, J. Managing Health and Well-Being in SMEs through an Adviceline: A Typology of Managerial Behaviours. Work Employment and Society. doi:10.1177/09500170211015113 |
| 1. Van de Velde, D., Eijkelkamp, A., Peersman, W., & De Vriendt, P. (2016). How competent are healthcare professionals in working according to a bio-psycho-social model in healthcare? The current status and validation of a scale. PLoS ONE, 11(10). doi:10.1371/journal.pone.0164018 |
| 1. Van der Gucht, N., & Lewis, K. (2015). Women's experiences of coping with pain during childbirth: A critical review of qualitative research. Midwifery, 31(3), 349-358. doi:10.1016/j.midw.2014.12.005 |
| 1. van Eijk-Hustings, Y., Kroese, M., Boonen, A., Bessems-Beks, M., & Landewé, R. (2015). Predictors for health improvement in patients with fibromyalgia: a 2-year follow-up study. Clinical Rheumatology, 34(1), 133-141. doi:10.1007/s10067-013-2371-7 |
| 1. Vassiliadou, I., Tolani, E., Ip, L., Smith, A., & Papachristou Nadal, I. (2020). Patient and public involvement in integrated psychosocial care. Journal of Integrated Care, 28(2), 135-143. doi:10.1108/JICA-06-2019-0027 |
| 1. Ventres, W. B., & Frankel, R. M. (2021). Personalizing the BioPsychoSocial Approach: “Add-Ons” and “Add-Ins” in Generalist Practice. Frontiers in psychiatry, 12. doi:10.3389/fpsyt.2021.716486 |
| 1. Voseckova, A., Truhlarova, Z., Levicka, J., Klimova, B., & Kuca, K. (2017). Application of salutogenic concept in social work with diabetic patients. Social Work in Health Care, 56(4), 244-259. doi:10.1080/00981389.2016.1265635 |
| 1. Wain, H. J., & Gabriel, G. M. (2007). Psychodynamic concepts inherent in a biopsychosocial model of care of traumatic injuries. Journal of the USAn Academy of Psychoanalysis and Dynamic Psychiatry, 35(4), 555-573. doi:10.1521/jaap.2007.35.4.555 |
| 1. Westheimer, J. M. M. A., Steinley-Bumgarner, M. M. A., & Brownson, C. P. (2008). Primary Care Providers' Perceptions of and Experiences With an Integrated Healthcare Model. Journal of USAn College Health, 57(1), 101-108. doi:http://dx.doi.org/10.3200/JACH.57.1.101-108 |
| 1. White, J., & Wills, J. (2011). What's the future for health promotion in England? The views of practitioners. Perspectives in Public Health, 131(1), 44-47. Retrieved from https://www.proquest.com/scholarly-journals/whats-future-health-promotion-england-views/docview/849563349/se-2?accountid=14680 |
| 1. Whittaker, K., & Cowley, S. (2003). Parenting support: Where does it fit with public health roles?: The Journal of the Health Visitors' Association. Community Practitioner, 76(3), 100. Retrieved from https://www.proquest.com/scholarly-journals/parenting-support-where-does-fit-with-public/docview/213337314/se-2?accountid=14680 |
| 1. Wildman, J., & Wildman, J. M. (2019). Combining health and outcomes beyond health in complex evaluations of complex interventions: Suggestions for economic evaluation. Value in Health, 22(5), 511-517. doi:10.1016/j.jval.2019.01.002 |
| 1. Wiley-Exley., E. (2007). Evaluations of community mental health care in low- and middle-income countries: A 10-year review of the literature. Social Science & Medicine, 64(6), 1231-1241. doi:http://dx.doi.org/10.1016/j.socscimed.2006.11.009 |
| 1. Williams, S. L., Haskard, K. B., & DiMatteo, M. R. (2007). The therapeutic effects of the physician-older patient relationship: effective communication with vulnerable older patients. Clinical interventions in aging, 2(3), 453-467. Retrieved from https://search.ebscohost.com/login.aspx?direct=true&AuthType=cookie,ip,shib,uid&db=cmedm&AN=18044195&site=ehost-live&scope=site&authtype=shib&custid=s8000044 |
| 1. Wilson, N. L., Malmberg, B., & Zarit, S. H. (1993). Group homes for people with dementia: A swedish example. Gerontologist, 33(5), 682-686. doi:10.1093/geront/33.5.682 |
| 1. Woodall, J., de Viggiani, N., Dixey, R., & South, J. (2014). Moving prison health promotion along: Towards an integrative framework for action to develop health promotion and tackle the social determinants of health. Criminal Justice Studies: A Critical Journal of Crime, Law & Society, 27(1), 114-132. Retrieved from https://search.ebscohost.com/login.aspx?direct=true&AuthType=cookie,ip,shib,uid&db=psyh&AN=2014-03343-008&site=ehost-live&scope=site&authtype=shib&custid=s8000044 |
| 1. Wright, J., Parry, J., & Scully, E. (2005). Institutionalizing policy-level health impact assessment in Europe: is coupling health impact assessment with strategic environmental assessment the next step forward? Bulletin of the World Health Organization, 83(6), 472-477. Retrieved from <Go to ISI>://WOS:000229851000014 |
| 1. Xiao, X., Song, H., Sang, T., Wu, Z., Xie, Y., & Yang, Q. (2021). Analysis of Real-World Implementation of the Biopsychosocial Approach to Healthcare: Evidence From a Combination of Qualitative and Quantitative Methods. Frontiers in psychiatry, 12. doi:10.3389/fpsyt.2021.725596 |
| 1. Yeung, E. W., Arewasikporn, A., & Zautra, A. J. (2012). Resilience and Chronic Pain. Journal of Social and Clinical Psychology, 31(6), 593-617. doi:http://dx.doi.org/10.1521/jscp.2012.31.6.593 |
| 1. Zittel, K. M., Lawrence, S., & Wodarski, J. S. (2002). Biopsychosocial model of health and healing: Implications for health social work practice. Journal of Human Behavior in the Social Environment, 5(1), 19-33. doi:10.1300/J137v05n01_02 |
